# Angiogenic Properties of Menstrual Stem Cells Are Impaired in Women with a History of Preeclampsia

**DOI:** 10.1155/2019/1916542

**Published:** 2019-01-27

**Authors:** Manuel Varas-Godoy, Stephanie Acuña-Gallardo, Sebastian Venegas-Duarte, Charlotte Hill, Albano Caceres-Verschae, Ornella Realini, Lara J. Monteiro, Gabriela Zavala, Maroun Khoury, Roberto Romero, Gregory Rice, Sebastian E. Illanes

**Affiliations:** ^1^Laboratory of Reproductive Biology, Center for Biomedical Research, Faculty of Medicine, Universidad de Los Andes, Santiago, Chile; ^2^Department of Obstetrics and Gynaecology, Faculty of Medicine, Universidad de los Andes, Santiago, Chile; ^3^Laboratory of Nano-Regenerative Medicine, Faculty of Medicine, Universidad de Los Andes, Santiago, Chile; ^4^Perinatology Research Branch, NICHD/NIH, Wayne State University, Detroit, MI, USA; ^5^Department of Obstetrics and Gynaecology, Clínica Davila, Santiago, Chile

## Abstract

Preeclampsia is a pregnancy-specific disorder defined by the new onset of hypertension and proteinuria after 20 weeks of gestation. Although its precise etiology is still unknown, there is evidence suggesting that it may be a consequence of impaired decidual and stromal cell function. Recently, a stem cell population derived from endometrial tissue and isolated from menstrual effluent called menstrual stem cells (MenSCs) has been identified. MenSCs exhibit important angiogenic and inflammatory properties that may contribute to both normal and pathological complications of implantation and placentation, including preeclampsia. We hypothesized that the angiogenic and inflammatory activity of MenSCs is altered in women who have a past history of preeclampsia and that this phenotype persists postpartum. The primary outcome measures were stromal progenitor cell formation, *in vitro* induction of endothelial tube formation, and release of proinflammatory cytokines. MenSCs obtained from women with a previous normal or preeclamptic pregnancy displayed similar phenotypic characteristics, tri-differentiation capacity, and proliferation. MenSCs derived from women who had preeclampsia on their previous pregnancy had reduced angiogenic capacity (~30% fewer junctions and nodes, *p* < 0.05) and stromal progenitor cell formation (<50% measured at a serial dilution of 1 : 10.000, *p* < 0.05) when compared to controls. *In vitro*, MenSCs obtained from patients with a history of preeclampsia expressed less endoglin and secreted less VEGF but more IL-6 than controls did. These data are consistent with the hypothesis that the angiogenic and inflammatory properties of MenSCs of women with a previous pregnancy complicated by preeclampsia have reduced angiogenic capacity and are more proinflammatory than those of MenSCs of women with a previous normal pregnancy. This altered phenotype of MenSCs observed following preeclampsia could either be present before the development of the pathology, predisposing the endometrial milieu to and consequently leading to limited vascular remodeling, or be a consequence of preeclampsia itself. The former may afford opportunity for targeted therapeutic intervention; the latter, a putative biomarker for future risk of pregnancy complications.

## 1. Introduction

Preeclampsia (PE) is a life-threatening vascular disorder of pregnancy due to a failing placenta that affects 3% to 5% of all pregnancies and is a major cause of maternal and perinatal morbidity and mortality [1]. PE is characterized by inadequate remodeling of uterine spiral arteries and reduced uteroplacental blood flow. These defects may be a consequence of shallow invasion by extravillous trophoblast cells [2–6], recruitment and function of maternal immune cells [7–11], endovascular trophoblast infiltration [2, 4, 12, 13], maturation of endometrial cells [14, 15], and decidual and stromal cell function [15–18], but most likely it could be due to a combination of all of these factors [19, 20].

Recently, endometrial cells have demonstrated to play a central role in both normal and abnormal early pregnancy development [21–31]. Notably, a growing body of evidence shows that defective endometrial cells and aberrant decidual response are implicated in the development of obstetrical syndromes such as PE [15, 32–34]. In fact, decidualization disorders have been detected at the time of delivery and have been proposed to persist years after the affected pregnancy [14, 15, 18]. Thus, not only does this support the idea that endometrial cells could reflect abnormal processes occurring in PE, during the pregnancy, but it also suggests that even years after pregnancy the abnormal phenotype could remain.

In the past decade, a population of mesenchymal stem cells (MSCs) with endometrial origin has been identified and characterized from human endometrial tissue and menstrual fluid [35, 36]. The properties of MSCs isolated from menstrual fluid, called also menstrual stem cells (MenSCs), have been extensively characterized [37–40]. Alcayaga-Miranda and colleagues showed that MenSCs display improved angiogenic properties, including VEGF secretion, in comparison with MSCs isolated from the bone marrow [40]. These data support the idea that MenSCs could reflect the status of the endometrium at the time of implantation and angiogenic changes occurring in normal and abnormal trophoblast invasion.

Although implantation and angiogenesis are both impaired in PE, the relation between the angiogenic properties of MSCs derived from the endometrium and the pathology of PE has not been evaluated. In this study, we characterized the phenotype of MenSCs isolated from women who previously developed PE (MenSCs-PE) in comparison with MenSCs isolated from healthy donors (MenSCs-C) in relation to their morphological, immunophenotypic, and functional characteristics. In a second phase, we evaluated the angiogenic properties of these cells by measuring their capacity of tube formation and secretion of the soluble factors, VEGF and endoglin, and cytokines that can modulate angiogenesis such as IL-6, IL-1*β*, and GM-CSF, among others.

## 2. Material and Methods

### 2.1. Study Design and Subjects

All human samples and cells used in this study were collected and harvested with the informed consent of the donor as approved by the ethical scientific committee of Clinica Davila and Universidad de los Andes. MenSCs were obtained from nine healthy donors (control) and six women who had PE on their previous pregnancies (9-12 months postpartum). Demographic characteristics were recorded. All women were using natural family planning, were not breastfeeding, had regular menses, and had not used contraceptives for at least 3 months before enrolment in the study.

### 2.2. Isolation and Culture of MenSCs

MenSCs were isolated as described previously [40]. Briefly, menstrual effluent was collected in a menstrual silicone cup (Mialuna, Santiago, Chile) during the first 48 h of the menstruation cycle and transferred into a 50 ml tube with 10 ml phosphate-buffered saline 1x (PBS) containing 0.25 mg/ml amphotericin B, 100 IU penicillin, 100 mg/ml streptomycin, and 2 mM ethylenediaminetetraacetic acid (EDTA) (Gibco, Thermo Fisher Scientific, MA, USA). MenSCs were separated from menstrual blood-mononuclear cells and other debris by Ficoll-Paque Plus density gradient (GE Healthcare, MI, USA) according to the manufacturer's instructions and washed in PBS. Subsequently, cells were cultured in high-glucose Dulbecco's modified Eagle's medium (DMEM, Gibco) supplemented with 1% penicillin/streptomycin (P/S), 1% amphotericin B, 1% glutamine (Gibco), and 15% fetal bovine serum (FBS) (HyClone, GE Healthcare) at 37°C, 5% CO_2_. Media were changed the day after to remove any nonadherent cells. All experiments were performed using MenSCs at early passages (P) P2 to P6. All cells tested negative for mycoplasma.

### 2.3. Phenotypic Characterization of MenSCs

For phenotypic characterization, cells were harvested, washed with cytometer buffer (PBS + 0.2% BSA + 0.01% sodium azide (Sigma-Aldrich, MI, USA)), and incubated with the specific labelled antibodies in cytometer buffer for 20 min at 4°C. Antibodies for human cell surface antigens CD14, CD44, CD90, CD105, CD73, CD45, CD34, HLA-ABC, HLA-DR, and CD146 were purchased from BD Biosciences, MD, USA, R&D Systems, MN, USA, and BioLegend, CA, USA. Matching isotype antibodies were used as negative controls in all experiments. In addition, LIVE/DEAD® Fixable dead cell stain kit (Invitrogen, CA, USA) was used to determine the viability of cells by flow cytometry according to the manufacturer's protocol. Data were collected using a fluorescence-activated cell sorting (FACS) Canto II flow cytometer (BD Biosciences) and analyzed on the FlowJo analysis software.

### 2.4. Tri-Differentiation Assay

MenSCs were induced to differentiation into chondrogenic, osteogenic, and adipogenic lineages using specific media over 21 days. For osteogenic differentiation, media were composed of DMEM high glucose (Corning, MA, USA) supplemented with FBS 5% (Gibco), 155 nM ascorbic acid (Sigma), 10 *μ*M *β*-glycerophosphate (Sigma-Aldrich), 0.1 *μ*M dexamethasone (Sigma-Aldrich), 2 mM L-glutamine (Gibco), and 100 U/ml penicillin-streptomycin. Differentiation was evaluated for genetic evaluation. Briefly, cells were lysed with TRIzol™ Reagent (Thermo Fisher), and RNA extraction was performed according to the manufacturer's instructions. 2 *μ*g of RNA was used for reverse transcription using the SuperScript II enzyme (Thermo Fisher), and cDNA was stored at –20°C until further analysis. qPCR was carried out using Brilliant II SYBR Green qPCR Master Mix (Stratagene, CA, USA) according to the manufacturer's instructions and amplified with qPCR System 3000X (Stratagene). *GAPDH* was used as a housekeeping gene for normalization. Primers' details are provided in Table 1.

### 2.5. Colony-Forming Unit Assay

To quantify the frequency of stromal progenitors, cells obtained after Ficoll centrifugation of the menstrual effluent were plated at a density of 100, 1,000, 10,000, and 100,000 nucleated cells/cm^2^ in DMEM. The medium was changed the next day to remove nonadherent cells. The frequency of progenitors was calculated following the extreme limiting dilution analysis (ELDA) method for comparing depleted and enriched populations in stem cells [40]. To quantify functional MSCs, MenSCs from both groups were evaluated for frequency of colony-forming units (CFU). CFU in passage 4 (P4) were evaluated in a serial dilution assay, where 25 to 250 cells per well were seeded in a 6-well plate (Falcon, Corning). After 14 days, colonies were stained with 0.5% crystal violet (Sigma-Aldrich) in 10% methanol for 20 min. Colonies formed by more than 50 cells were counted under a light microscope at low magnification. Results were expressed as CFU/number of cells plated.

### 2.6. Proliferation Assay

MenSCs were cultured at 1,000 cells/cm^2^ in 24-well plates (Falcon, Corning) in complete DMEM (10% FBS, 1% penicillin-streptomycin). Cell proliferation and viability were determined at days 3, 6, and 9 by the SRB Assay (BioVision, CA, USA) and by spectrophotometric quantification (absorbance, 492 nm) according to the manufacturer's instructions.

### 2.7. Decidualization Recapitulated *In Vitro*

MenSCs were cultured under an endometrial hormonal milieu. Briefly, 120,000 MenSCs were cultured in a 6-well plate with complete DMEM without phenol red (Gibco) and exposed to 5% oxygen conditions (5% O_2_). 24 h after, the medium was replaced with DMEM without phenol red and 10% FBS, treated with active carbon and 1% penicillin-streptomycin, supplemented with estrogen (E2; 213 pg/ml, Sigma-Aldrich, MI, USA), and cultured at 5% O_2_ for 24 h. The medium was replaced for the same media described above but supplemented with both estrogen and progesterone (E2; 146 pg/ml, P4; 11 ng/ml, respectively, Sigma-Aldrich, MI, USA) and cultured at 5% O_2_. 24 h later, conditioned media were harvested, centrifuged at 500 g for 5 min, and stored at -80°C for subsequent analysis: angiogenesis assay, ELISA, and protein solution array assays (Luminex Corporation, TX, USA). MenSC cellular lysates were collected for Western blot analysis.

### 2.8. Angiogenesis Assay

Tube formation in Matrigel (Corning) was used as a functional assay to evaluate the angiogenic properties of MenSCs. Briefly, human umbilical vein endothelial cells (HUVECs) were seeded in 48-well plates precoated with 70 *μ*l of growth factor reduced Matrigel (Corning, Manassas, VA, USA) at a density of 4 × 10^4^ cells/well in the conditioned medium harvested previously. After 16 hours, the tube formation was examined by phase-contrast and the images were captured using an Olympus U-RFL-T camera. Junctions, nodes, meshes, and branches of tubes were analyzed using the ImageJ Angiogenesis Analyzer software.

### 2.9. Western Blot

Protein lysates were extracted from MenSCs exposed to endometrial conditions using RIPA buffer. Protein concentration was measured using the Qubit Protein Assay kit (Thermo Fisher Scientific), and 5 *μ*g of proteins was separated on a 12% polyacrylamide gel and transferred to polyvinylidene difluoride membrane (PVDF, Thermo Scientific). The membrane was blocked with 5% skimmed milk in PBS 1x Tween 0.1% (PBS 10x, Corning; Tween 20, Winkler) for 1 h at room temperature and then incubated overnight at 4°C with anti-VEGF (cat# ab183100, 1 : 1.000, Abcam, Cambridge, United Kingdom), anti-endoglin (cat# ab137389, 1 : 1.000, Abcam, Cambridge, United Kingdom), or *β*-actin (cat# Ab183100, 1 : 1,000, Abcam, Cambridge, United Kingdom). After overnight incubation, membranes were washed and exposed for 2 h to the anti-rabbit secondary antibody (cat# 474-1506, 1 : 10,000, Kirkegaard & Perry Lab Inc., Gaithersburg, MD) to VEGF and endoglin and anti-mouse secondary antibody (cat# ab6276, 1 : 10,000, Kirkegaard & Perry Lab Inc., Gaithersburg, MD) to *β*-actin. The signal was detected with horseradish peroxidase-linked anti-rabbit or anti-mouse (KPL), and chemiluminescence was visualized using the ECL detection system (Thermo Fisher Scientific, Waltham, MA).

### 2.10. Quantification of Angiogenic Secreted Factors by ELISA

The conditioned medium harvested from MenSCs cultured under an endometrial hormonal microenvironment was concentrated using 10 kDa Amicon Ultra-4 filters (Millipore, St. Charles, MO, USA), and the protein concentration of the concentrated conditioned medium was measured using the Qubit Protein Assay kit (Thermo Fisher Scientific). VEGF, soluble endoglin (sEng), soluble fms-like tyrosine kinase-1 (sFlt1), angiopoietin 1, and angiopoietin 2 concentrations were detected using ELISA kits (VEGF, cat# DY293B; endoglin, cat# DY1097; sFlt1, cat# DVR100C; angiopoietin 1, cat# DANG10; and angiopoietin-2, cat# DANG20; R&D Systems, Minneapolis, MN, USA) according to the manufacturer's protocol.

### 2.11. Proinflammatory Cytokine Profile

The secreted cytokine profile was determined using a commercial 11-multiplex fluorescent bead-based immunoassay (R&D Systems). The concentration of the following cytokines was determined: IL-8, IFN-*γ*, IL-1*β*, IL-12p70, IL-2, TNF-*α*, GM-CSF, IL-1*α*, IL-10, IL-17A, and IL-6. The same concentrated conditioned medium from the ELISA analysis was used. Briefly, 50 *μ*l of the concentrated conditioned medium was incubated with diluted bead cocktail for 2 h, with constant shaking at room temperature. The beads were washed and incubated with detection antibodies for 1 h with constant shaking at room temperature. The beads were washed, and streptavidin-PE was added and allowed to incubate for 30 min with constant shaking at room temperature (in the dark). The beads were then washed and suspended in 100 *μ*l of wash buffer and incubated for 2 min on the shaker prior to reading. Fluorescence was determined on the MAGPIX® System (Thermo Fisher Scientific), and the cytokine concentrations for each sample were extrapolated based on standards utilizing Milliplex Analyst 5.1 software (Millipore) with a 4-parameter logistic curve (4-PL).

### 2.12. Statistical Analyses

Statistical analyses were performed using the *t*-test, and statistical significance was set at *p* < 0.05. Data was analyzed using the GraphPad Prism 6.0 program (GraphPad Software, La Jolla, CA, USA).

## 3. Results

The demographic characteristics of the PE (case group) and control groups are presented in Table 2. No significant differences were observed in age, weight, and height in both groups. BMI was higher in the women with a history of PE in comparison with healthy controls (*p* = 0.0496). In terms of obstetric history, the healthy women (control group) were a mixture between nulliparous (4) and multiparous (5).

**Figure 1:**
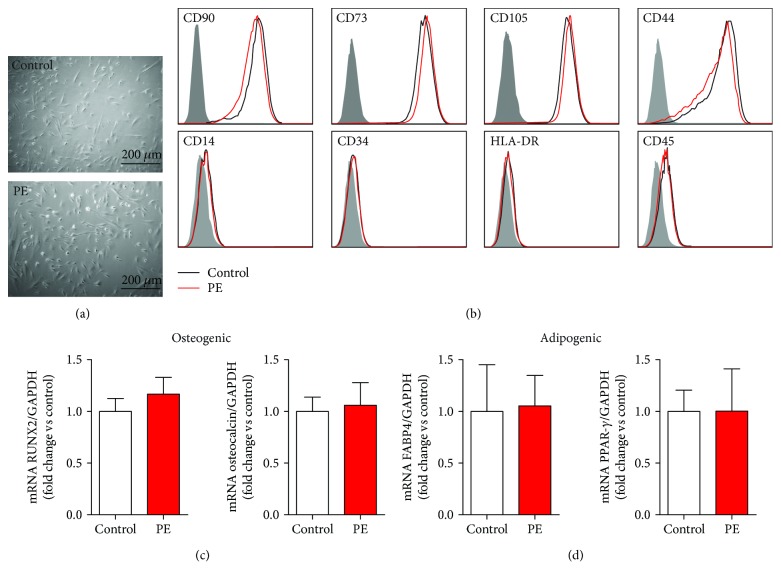
Morphology, phenotypic markers, and multilineage capacities of MenSCs-PE. (a) Fibroblast-like morphology of MenSCs isolated from healthy women (control) and women with a previous preeclampsia (PE). MenSCs from PE showed the same morphology as MenSCs isolated from control. (b) Phenotypic characterization of MSC surface markers in MenSCs isolated from women with a history of PE (red) and control (black) and their respective isotype control (gray); the cells were analyzed by flow cytometry. All MenSCs from both groups were positive for CD90, CD73, CD105 (endoglin), and CD44 and negative for CD14, CD34, HLA-DR, and CD45. (c) Osteogenic differentiation of MenSCs isolated from healthy control women and women who developed PE was evaluated by measuring mRNA expression of RUNX2 and osteocalcin by qPCR. (d) Adipogenic differentiation of MenSCs isolated from healthy control women and women who developed PE was evaluated by measuring FABP4 and PPAR-*γ* by qRT-PCR. Results are mean + SEM (standard error of the mean). Statistical analysis was performed using Student's *t*-tests.

For case and control groups, MenSC morphology, phenotypic characteristics, and potential to differentiate into adipocytes, osteoblasts, and chondrocytes were compared. Both cases and controls displayed similar fibroblast-like morphology (Figure 1(a)); expression of the classical MSC positive markers CD90, CD73, CD105, and CD44; absence of the negative markers CD14, CD34, CD45, and HLA-DR (Figure 1(b)); and capacity to differentiate into osteogenic, adipogenic, (Figures 1(c) and 1(d)), and chondrogenic lineages (Supplementary Figure 1). No difference was observed in the expression of pluripotency genes OCT4, SOX2, and NANOG (Supplementary Figure 2).

**Figure 2:**
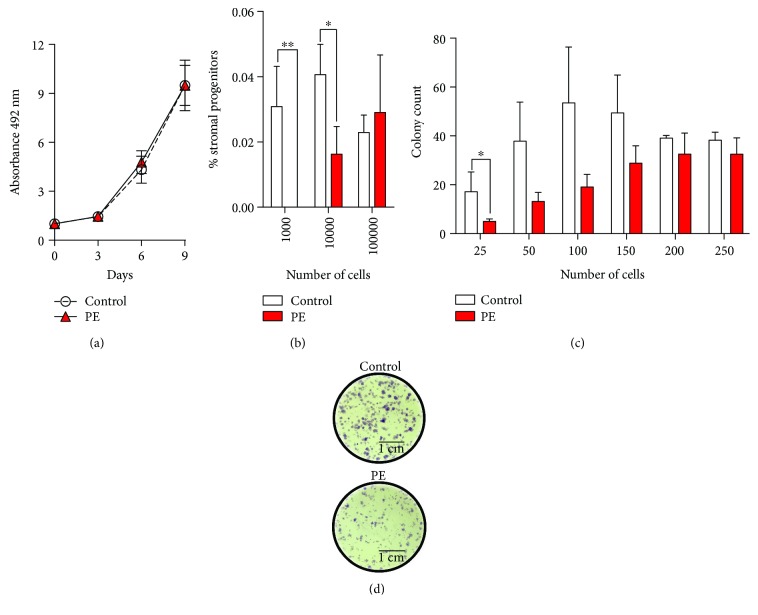
Proliferative and clonogenic potential of MenSCs-PE. (a) Proliferative capacity of MenSCs isolated from healthy women (control) and women who had a past history of preeclampsia (PE). Cell proliferation was evaluated at days 3, 6, and 9 by the SRB assay. MenSCs-PE showed a similar proliferation rate when compared to MenSCs isolated from healthy women. (b) Stromal progenitor capacity; serial dilutions of a defined number of cells in passage 0 (P0) were cultured, and the potential to form stromal progenitors was evaluated. MenSCs-PE show less initial frequency of stromal progenitors than do MenSCs from control women. (c) CFU capacity; serial dilutions of a defined number of cells in passage 4 (P4) were cultured, and the potential to form CFU was evaluated. MenSCs-PE show less frequency of CFU capacity after cell expansion in low dilutions. (d) Representative images of CFU after cell expansion in low dilutions of MenSCs-PE and healthy women (control) cultured at P4. Results are the mean + SEM (standard error of the mean). Statistical analysis was performed using Student's *t*-tests. ^∗^ *p* < 0.05 and ^∗∗^ *p* < 0.01 significant.

To compare functional properties of MenSCs, the proliferative and clonogenic potential of these cells was assessed. The proliferation rate was similar in both case and control groups (Figure 2(a)). The initial clonogenic capacity of MenSCs isolated from women with a previous pregnancy complicated by preeclampsia, however, was reduced when compared to controls (Figure 2(b)), with reduced initial frequency of mesenchymal progenitors (PE: 1 in 874 with 95% confidence interval (CI) of 1/471 to 1/1620; control: 1 in 358 with 95% confidence interval (CI) of 1/227 to 1/566) (Supplementary Figure 3). The frequency of CFU after cell expansion in high dilutions was also reduced in MenSCs isolated from women with a history of preeclampsia (Figures 2(c) and 2(d)). We have also evaluated the gene expression of metalloproteinase-3 (MMP-3), a gene involved in MSC migration, and we observed a significant decrease in the MMP-3 mRNA expression in the MenSCs derived from women with a past history of PE (Supplementary Figure 4).

Since the human uterus is exposed to oxygen tensions of 2-5% [41] and varying hormonal profiles throughout the menstrual cycle [42], MenSC angiogenic activity was assessed under conditions that recapitulated the decidualization microenvironment of the human menstrual cycle *in vitro*. The implantation window during a normal menstrual cycle is characterized by increased VEGF and leukemia inhibitory factor (LIF) expression. This expression profile was recapitulated in MenSCs from healthy donors incubated *in vitro* under an environment that mimics the receptive phase (Supplementary Figure 5).

**Figure 3:**
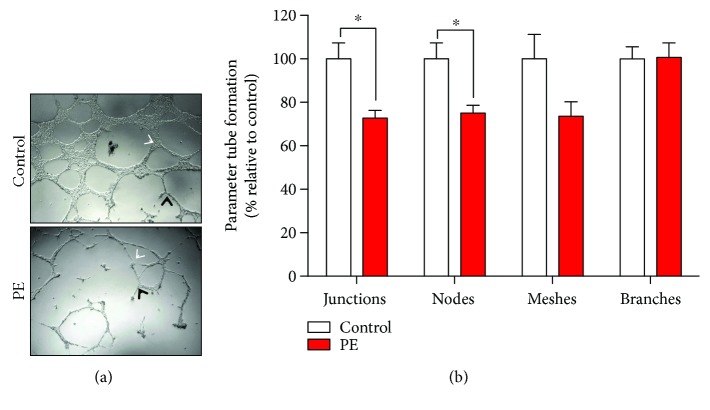
Reduced tube formation capacity on endothelial cells under MenSCs-PE conditioned media. (a) Representative images of tubule structure formation of HUVECs incubated with conditioned medium from MenSCs isolated from healthy women (control) and women with a previous history of preeclampsia (PE), cultured under decidualization conditions of the uterine-endometrial microenvironment. Black arrows represent nodes, and white arrows represent junctions. (b) Quantification of different tube formation parameters (junctions, nodes, meshes, and branches) from MenSCs from women with previous PE showed lower capacity of tube formation than that from MenSCs isolated from control women represented in a significant decrease in junctions and nodes. Results are mean + SEM (standard error of the mean). Statistical analysis was performed using Student's *t*-tests. ^∗^ *p* < 0.05 significant.

MenSCs isolated from women with a previous pregnancy complicated by PE have reduced angiogenic capacity compared to controls (Figure 3(a)). The quantification of different parameters of tube formation showed a significant decrease of approximately 30% in junctions and nodes (Figure 3(b)). No significant differences were identified in meshes or branches (Figure 3(b)).

**Figure 4:**
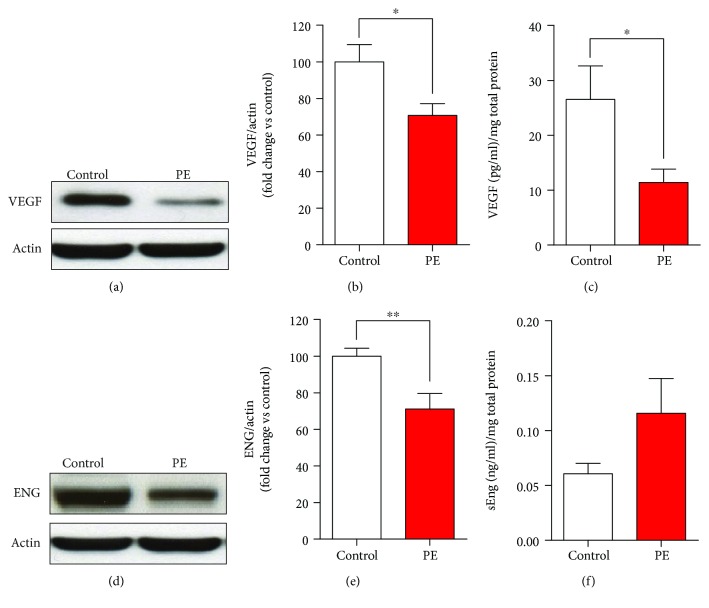
Reduced expression and secretion of VEGF and reduced expression of endoglin on MenSCs-PE. (a) Representative VEGF Western blot of MenSCs isolated from healthy women (control) and women who developed preeclampsia (PE) cultured under conditions that recapitulate the decidualization *in vitro*. (b) Semiquantification of VEGF by Western blot of MenSCs from PE showed a significant lower protein expression of VEGF than that of MenSCs isolated from control women. (c) VEGF secretion measured by ELISA analysis of MenSCs from control and PE; MenSCs from women with past PE had significantly reduced secretion of VEGF compared to MenSCs isolated from control women. (d) Representative endoglin Western blot of MenSCs from control and PE cultured under conditions that recapitulate the decidualization *in vitro*. (e) Semiquantification of endoglin protein expression demonstrated significantly lower protein expression of endoglin in MenSCs from women with past PE compared to MenSCs isolated from control women. (f) Analysis of endoglin secretion demonstrated no significant difference in endoglin secretion in MenSCs from control and PE. Results are mean + SEM (standard error of the mean). Statistical analysis was performed using Student's *t*-tests. ^∗^ *p* < 0.05 and ^∗∗^ *p* < 0.01 significant.

**Figure 5:**
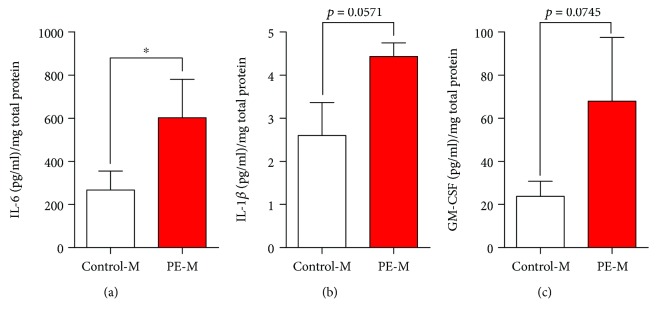
Increased secretion of IL-6 on MenSCs-PE. (a) IL-6, (b) IL-1*β*, and (c) GM-CSF secretion measured by multiplex fluorescent bead-based immunoassay analysis of MenSCs from control and PE; MenSCs from women with past PE had significantly increased secretion of IL-6 compared to MenSCs isolated from control women. Results are mean + SEM (standard error of the mean). Statistical analysis was performed using Student's *t*-tests. ^∗^ *p* < 0.05.

VEGF protein expression (Figures 4(a) and 4(b)) and secretion to the culture media (Figure 4(c) and Table 3) were reduced in cases compared to controls. MenSCs from the PE group expressed less endoglin (Figures 4(d) and 4(e)); however, no statistically significant change was identified in secretion (Figure 4(f) and Table 3). Similarly, no statistically significant changes were identified in sFlt1, angiopoietin-1, or angiopoietin-2 concentrations (Table 3). IL-6 secretion from MenSCs from the PE group was 2.3-fold greater than that observed for controls (Figure 5 and Table 4). No significant differences were observed in the secretion levels of IL-1*β*, GM-CSF, TNF-*α*, IL-8, IL-10, IFN-*γ*, IL-1*α*, IL-17A, IL-2, and IL-12p70 (Figure 5 and Table 4).

Since decidualization has been related to the development of PE, we evaluated the mRNA expression of genes involved in the decidualization process, such as IGFBP-1 and TGF-*β* [43, 44], but we did not observe a reduction in the mRNA expression of both genes on MenSCs from women with a history of PE (Supplementary Figure 6).

## 4. Discussion

The menstrual fluid is generally considered to represent a combination of blood and desquamated cells from the endometrium, many of which are nonviable or in a senescent state. A subpopulation of these cells displays stem cell properties, including angiogenic activity. The data obtained in this study suggest that these cells retain phenotypic variations postpartum that may be associated with and account for the abnormal implantation process observed in preeclampsia. MenSCs obtained from women with a previous preeclamptic pregnancy express less VEGF and membrane-bound endoglin, induce less endothelial cell tube formation, and release more IL-6 than do cells obtained from women with a normotensive previous pregnancy or from women that were never pregnant. The observed phenotypic variations are constituent with the pathophysiology of PE.

Different studies have shown the importance of endometrial cells in the natural selection of human embryos in the process of implantation [27, 45] and as revealed by a recent study that endometrial stromal cell secretome is associated with embryo implantation failure [46]. VEGF is one of the secreted factors contributing to successful implantation [47, 48] due to its control of the angiogenic process that is tightly regulated during the menstrual cycle [49–51]. Moreover, mice treated with the angiogenic inhibitor AGM-1470 present impaired endometrial maturation and decidualization affecting the embryo development and implantation [52]. Likewise, administration of VEGF antibodies affected the endometrial vascular permeability in rats, another event associated with implantation [53]. According to our results, VEGF expression on MenSC-PE is decreased, even one year after pregnancy, highlighting the potential role of these cells in the abnormal angiogenic process of implantation of patients with a history of PE.

Decidualization induces a switch in the secretion of different secreted factors around the perivascular niche to promote trophoblast invasion and to regulate immune response. Decidualization defects have been described to be involved in the development of PE [14, 15, 18]. In fact, it has been proposed that analysis of the decidual response could determine the risk of placental disorders in women [54]. Moreover, in PE, decidualization disorders have been detected at the time of delivery and may persist years after the affected pregnancy. Human endometrial stromal cells (hESC) obtained from nonpregnant women with a history of severe PE do not decidualize *in vitro* in response to cAMP and medroxyprogesterone acetate and release significantly less decidualization markers (prolactin and IGFBP-1) than do those derived from women with a previous normal pregnancy. Similarly, decidual cells (from the decidua basalis and parietalis) obtained from pregnant women with severe PE do not express decidualization markers (IGFBP-1 and prolactin) in comparison with a gestational age-matched control group and do not redecidualize *in vitro* [18]. Importantly, circulating concentrations of IGFBP-1 are lower in pregnant patients who subsequently develop PE than in those with a normal pregnancy outcome [55, 56]. Furthermore, decidual cell-conditioned media from patients with severe PE do not promote cytotrophoblast invasion; however, this defect is corrected by the addition of prolactin and IGFBP-1 [18]. Our results showed a decrease in the IGFBP-1 on MenSC-PE, establishing that these anomalies are related not only to impaired decidualization but also to an impaired angiogenesis, highlighting the potential importance of the cells present in the decidua in the pathogenesis of preeclampsia.

Venkatesha and collaborators showed that soluble endoglin (sEng) plays an important role in the pathogenesis of PE affecting the placental vascular function [57]. During normal pregnancy, sEng liberation is inhibited from the placenta and endothelium through proinflammatory cytokines such as TNF-*α* and IFN-*γ*, but in a preeclamptic pregnancy sEng is increased along with sFlt-1 before the onset of the clinical symptoms of preeclampsia [58, 59]. Although previous studies have investigated the mechanism of sEng secretion and function of membrane-bound endoglin on endothelial cells and angiogenesis [57], there is contradictory information about the tissue origin of circulating sEng during preeclampsia. A recent *in vitro* study confirms that pAkt/Akt is reduced in preeclamptic placentas and suggests that sEng originates from endothelial cells and not from the placenta cells [60]. Although this study provided valuable data, it was unable to demonstrate that this reduction contributes to excessive sEng release from placenta. Recently, Chadchan et al. explored the role of endoglin in the uterine receptivity for embryo implantation in a murine model and identified increased endoglin expression in the endometrium during the implantation window and a reduced implantation index following endoglin knockdown [61]. Our results highlight the potential importance of the membrane-bound endoglin during the implantation window and the impairment of its expression in patients with history of PE. In addition, our data are consistent with an endometrial stem cell contribution to increased plasma sEng concentrations during preeclampsia.

In addition to changes in Eng on MenSC-PE, we also observed an increased secretion of IL-6 from these cells. Indeed, PE has been associated with an immune imbalance which induces a systemic inflammation [62]. In patients with PE, the decidua basalis shows abnormal T-regulatory cell function, as well as changes in macrophage number and phenotype, and an increase in proinflammatory cytokine expression, including IL-6 [63–65]. Interestingly, IL-6 has been implicated to stimulate a defective angiogenesis in tumor models [66]. Moreover, IL-6 has also been described as a cytokine with endometrial function and with a role in implantation [67]. Our results are consistent with the idea that mesenchymal stem cells present in the decidua could be contributing to the development of PE by releasing factors such as IL-6. Future studies will be needed in order to clarify how MenSCs could contribute to the preeclampsia pathogenesis, but preliminarily, our results suggest a role for these cells in mediating both angiogenesis impairment and potentially inflammation.

## 5. Conclusions

In summary, previous studies suggest that endometrial stromal cells obtained from individual patients closely phenocopy the decidualization process. Menstrual fluid contains a subpopulation of mesenchymal stem cells of endometrial origin. Menstrual stem cells from women who had a previous preeclampsia display different angiogenic and inflammatory properties than do those obtained from patients who had a previous normal pregnancy. These data may be useful in the development of predictive models to identify patients at risk of preeclampsia in the preconceptional stage.

## Figures and Tables

**Table 1:** Tri-differentiation primers.

Gene	Lineage	Primer
*RunX2* F	Osteogenic	CGG AAT GCC TCT GCT GTT AT
*RunX2 R*	Osteogenic	TTC CCG AGG TCC ATC TAC TG
*Osteocalcin F*	Osteogenic	GGC GCT ACC TGT ATC AAT GG
*Osteocalcin R*	Osteogenic	TCA GCC AAC TCG TCA CAG TC
*FABP4* F	Adipogenic	ATG GGA TGG AAA ATC AAC CA
*FABP4 R*	Adipogenic	GTG GAA GTG ACG CCT TTC AT
*PPAR-γ F*	Adipogenic	ATG GAG TCC ACG AGA TCA TT
*PPAR-γ R*	Adipogenic	CGC AGG CTC TTT AGA AAC TC
*GAPDH F*	Housekeeping	CAG CCT CAA GAT CAT CAG CA
*GAPDH R*	Housekeeping	CAT GAG TCC TTC CAC GAT AC

**Table 2:** Demographic characteristics.

Characteristics	Control (*n* = 9)	PE (*n* = 6)	*p* value
Age (years), median (IQ)	29.00 (28.00-33.00)	30.50 (26.75-34.25)	0.9319
Weight (kg), median (IQ)	60.00 (53.50-68.00)	73.50 (59.00-83.75)	0.0939
Height (mt), median (IQ)	1.650 (1.610-1.690)	1.605 (1.573-1.658)	0.2302
BMI (kg/cm^2^), median (IQ)	22.00 (19.40-24.80)	27.30 (23.18-33.68)	0.0496^∗^
Nullipara, *n* (%)	4 (44.4)	0 (0)	
Miscarriages, *n* (%)	1 (11.1)	1 (16.7)	
Early PE, *n* (%)		4 (66.7)	
Late PE, *n* (%)		2 (33.3)	
Early PE (weeks), median (IQ)		27.5 (24.5-27.5)	
Late PE, (weeks) median (IQ)		37.1 (36.4-37.7)	

Abbreviations: kg: kilograms; mt: meters; BMI: body mass index (kilograms/square meters). Results are median ± IQ (interquartile). Statistical analysis was performed using Student's *t*-tests. ^∗^ *p* < 0.05, significant.

**Table 3:** Levels of angiogenic factors secreted from MenSCs.

Characteristics	Control (*n* = 9)	PE (*n* = 6)	*p* value
VEGF (pg/ml)/mg total protein, median (IQ)	20.05 (13.70-45.18)	11.40 (5.438-17.60)	0.0233^∗^
sEng (ng/ml)/mg total protein, median (IQ)	0.066 (0.042-0.082)	0.088 (0.061-0.1845)	0.0637
sFlt1 (pg/ml)/mg total protein, median (IQ)	81.38 (30.43-117.2)	91.36 (20.39-281.3)	0.3826
Angiopoietin-1 (pg/ml)/mg total protein, median (IQ)	103.3 (28.99-168.9)	136.3 (48.26-239.7)	0.2264
Angiopoietin-2 (pg/ml)/mg total protein, median (IQ)	59.75 (40.76-128.9)	61.34 (37.49-127.7)	0.3275

Abbreviations: VEGF: vascular endothelial growth factor; sEng: soluble endoglin; sFlt1: soluble fms-like tyrosine kinase-1; pg: picograms; ng: nanograms; ml: milliliters; mg: milligrams. Results are median ± IQ (interquartile). Statistical analysis was performed using Student's *t*-tests. ^∗^ *p* < 0.05, significant.

**Table 4:** Levels of proinflammatory cytokines secreted from MenSCs.

Characteristics	Control (*n* = 9)	PE (*n* = 6)	*p* value
IL-8 (pg/ml)/mg total protein, median (IQ)	1359 (424.2-1423)	1355 (1126-1380)	0.4645
IFN-*γ* (pg/ml)/mg total protein, median (IQ)	4.913 (2.613-6.524)	5.465 (3.825-7.933)	0.2757
IL-1*β* (pg/ml)/mg total protein, median (IQ)	2.857 (0.697-3.694)	4.418 (3.862-5.020)	0.0571
IL-12p70 (pg/ml)/mg total protein, median (IQ)	7.911 (3.598-10.04)	9.369 (5.741-12.16)	0.2537
IL-2 (pg/ml)/mg total protein, median (IQ)	6.623 (5.346-7.394)	6.882 (4.991-9.218)	0.3247
TNF-*α* (pg/ml)/mg total protein, median (IQ)	1.178 (0.389-1.699)	1.296 (0.855-2.405)	0.2428
GM-CSF (pg/ml)/mg total protein, median (IQ)	17.55 (9.24-37.64)	34.20 (22.45-130.3)	0.0745
IL-1*α* (pg/ml)/mg total protein, median (IQ)	2.402 (1.183-3.586)	2.619 (1.581-5.668)	0.3423
IL-10 (pg/ml)/mg total protein, median (IQ)	0.336 (0.209-0.366)	0.274 (0.220-0.375)	0.4382
IL-17A (pg/ml)/mg total protein, median (IQ)	1.095 (0.619-1.363)	1.370 (0.753-1.630)	0.2194
IL-6 (pg/ml)/mg total protein, median (IQ)	228.6 (23.40-450.8)	349.8 (299.8-1032)	0.0145^∗^

Abbreviations: pg: picograms; ml: milliliters; mg: milligrams. Results are median ± IQ (interquartile). Statistical analysis was performed using Student's *t*-tests. ^∗^ *p* < 0.05, significant.

## Data Availability

The data used to support the findings of this study are included within the article and within the supplementary information file(s).

## Supplementary Materials

Supplementary Materials Supplementary Figures_Final: file with the legends of each Supplementary Figures. Supplementary Material_Final: file with all the methods related to the experiments performed for the Supplementary Figures. Supplementary Figure 1, 2, 3, 4, 5 and 6: supplementary figures in EPS format. The description of each figure is in the Supplementary Figures_Final file.
