# Supplementary material for: Angiogenic Properties of Menstrual Stem Cells Are Impaired in Women with a History of Preeclampsia
Source: Stem Cells Int. 2019 Jan 27;2019:1916542. doi: 10.1155/2019/1916542 (PMC6369467; doi:10.1155/2019/1916542)

Supplementary Materials

Chondrogenic differentiation

MenSC were induced to differentiation into condrogenic, osteogenic and adipogenic lineages using specific media during 21 days. For osteogenic differentiation, media was composed of DMEM high glucose (15018, Corning) supplemented with FBS 5% (10437028, Gibco), 155 nM ascorbic acid (49752, Sigma), 10 µM β-glycerophosphate (G9422, Sigma), 0.1 µM Dexamethasone (D4902, Sigma), 2mM L-Glutamine (25005, Gibco) and 100 U/ml Penicillin-Streptomycin. Differentiation was evaluated with Alizarin Red S stain. Adipogenic differentiation was induced with StemPro® Adipogenesis Differentiation Kit (A1007001, Thermo Fisher) and lipids drops were dyed with Oil Red O. Condrogenic differentiation was induced with StemPro™ Chondrogenesis Differentiation Kit (Thermo Fisher, A1007101) according to manufacturer instructions. Proteoglycans formation was dyed with Safranin O stain.

Colony forming unit assay

To quantify the frequency of stromal progenitors, cells obtained after Ficoll centrifugation of the menstrual effluent were resuspended in DMEM and plated at a density of 100, 1,000, 10,000 and 100,000 nucleated cells/cm^2. The medium was changed the next day to remove non-adherent cells. The frequency of progenitors was calculated by counting the number of CFU-F in each determined dilution and following the Extreme limiting dilution analysis (ELDA) method for comparing depleted and enriched populations in stem cells.

Quantification of VEGF by ELISA

MenSCs were cultured under standard conditions (21% O_2, without hormones) or endometrial hormonal conditions. Briefly, MenSCs were supplemented with estrogen (E2; 213 pg/ml, Sigma-Aldrich, MI, USA), and cultured at 5% O2 for 24 h. Medium was replaced for the same media described above but supplemented with both estrogen and progesterone (E2; 146 pg/ml, P4; 11 ng/ml, respectively, Sigma-Aldrich, MI, USA) and cultured at 5% O_2. After 24 h conditioned medium was harvested, centrifuged at 500 g for 5 min, concentrated using 10 kDa filters Amicon Ultra-4 (Millipore, St Charles, MO, USA), and stored at −80°C for subsequent analysis. Protein concentration of the concentrated conditioned medium was measured using Qubit Protein Assay kit (ThermoFisher Scientific). VEGF concentrations was detected using ELISA kits (VEGF; cat# DY293B, R&D Systems, Minneapolis, MN, USA) according to the manufacturer's protocol.

mRNA expression

MenSCs cultured control or decidualization conditions for LIF, and MenSCs derived from PE or control for Nanog, Sox2, Oct4, MMP-3, IGFBP-1 and TGF-β were lysed with TRIzol™

Reagent (Thermo Fisher) and RNA extraction was performed according to manufacturer instructions. 500 ng of total RNA were used for reverse transcription using the Superscript II enzyme (Thermo Fisher) and cDNA was stored at –20 °C until further analysis. LIF mRNA expression from both groups was evaluated by qPCR using Brilliant II SYBR Green qPCR Master Mix (Stratagene, CA, USA) according to manufacturer's instructions and amplified with qPCR System 3000X (Stratagene). TBP was used as a housekeeping gene for normalization. The primers used were:

**Gene**	**Forward**	**Reverse**
LIF	GTTTCCTCCAAGGCCCTCT	TGTTTCCAGTGCAGAACCAA
Nanog	CATGAGTGTGGATCCAGCTTG	CCTGAATAAGCAGATCCATGG
Sox2	AGCTACAGCATGATGCAGGA	GAGTAGGACATGCTGTAGGT
Oct4	AGGTATTCAGCCAAACGACCA	TCGATACTGGTTCGCTTTCTC
MMP-3	CTGGACTCCGACACTCTGGA	CAGGAAAGGTTCTGAAGTGACC
IGFBP-1	CCTATGATGGCTCGAAGGCT	TCCTGATGTCTCCTGTGCCT
TGF-β	CGACTCGCCAGAGTGGTTAT	CTAAGGCGAAAGCCCTCAAT
TBP	CACGAACCACGGCACTGAT	CACGAACCACGGCACTGAT

Supplementary Figures

Supplementary figure legend 1: Osteogenic, chondrogenic, and adipogenic differentiation potential in MensSC cultures from healthy and preeclamptic women (PE). Control and stimulated cells were stained with specific dyes in order to determine differentiation. Osteogenic, chondrogenic, and adipogenic differentiation was evaluated with Alizarin Red O, Safranin O, and Red Oil O respectively in A, D, G) non-treated cells, B, E, H) control and C, F, I) preeclamptic MenSCs.

Supplementary figure legend 2: MenSCs derived from women with history of preeclampsia (PE) show similar levels of Nanog, Sox2, and Oct4 mRNA expression in comparison with MenSCs derived from healthy donors (control).

Supplementary figure legend 3: Limiting dilution analyses of MenSCs define the frequency of progenitor's cells. Mononuclear cells obtained after the ficoll centrifugation of the menstrual blood were resuspended in DMEM and plated at a density of 100; 1,000; 10,000, and 100,000 nucleated cells/cm^2. Media were changed the next day to wash non adherent cells. The frequency of progenitors was calculated by counting the number of CFU-F in each determined dilution and following the Extreme limiting dilution analysis (ELDA) method for comparing depleted and enriched populations in stem cells. Group A: control, Group B: PE.

Supplementary figure legend 4: MenSCs derived from women with history of preeclampsia (PE) show lower levesl of MMP-3 mRNA expression in comparison with MenSCs derived from healthy donors (control).

Supplementary figure legend 5: MenSCs under decidualization condition show higher secretion of VEGF and higher LIF mRNA expression. A) VEGF secretion measured by ELISA analysis of MenSCs under decidualization condition (Dc) versus none decidualization conditions (control). B) LIF mRNA expression measured by qPCR of MenSCs under decidualization condition (Dc) versus none decidualization conditions (control).

Supplementary figure legend 6: MenSCs derived from women with history of preeclampsia (PE) show similar levels of IGFBP-1 and TGF-β mRNA expression in comparison with MenSCs derived from healthy donors (control).

Supplementary Figure 1

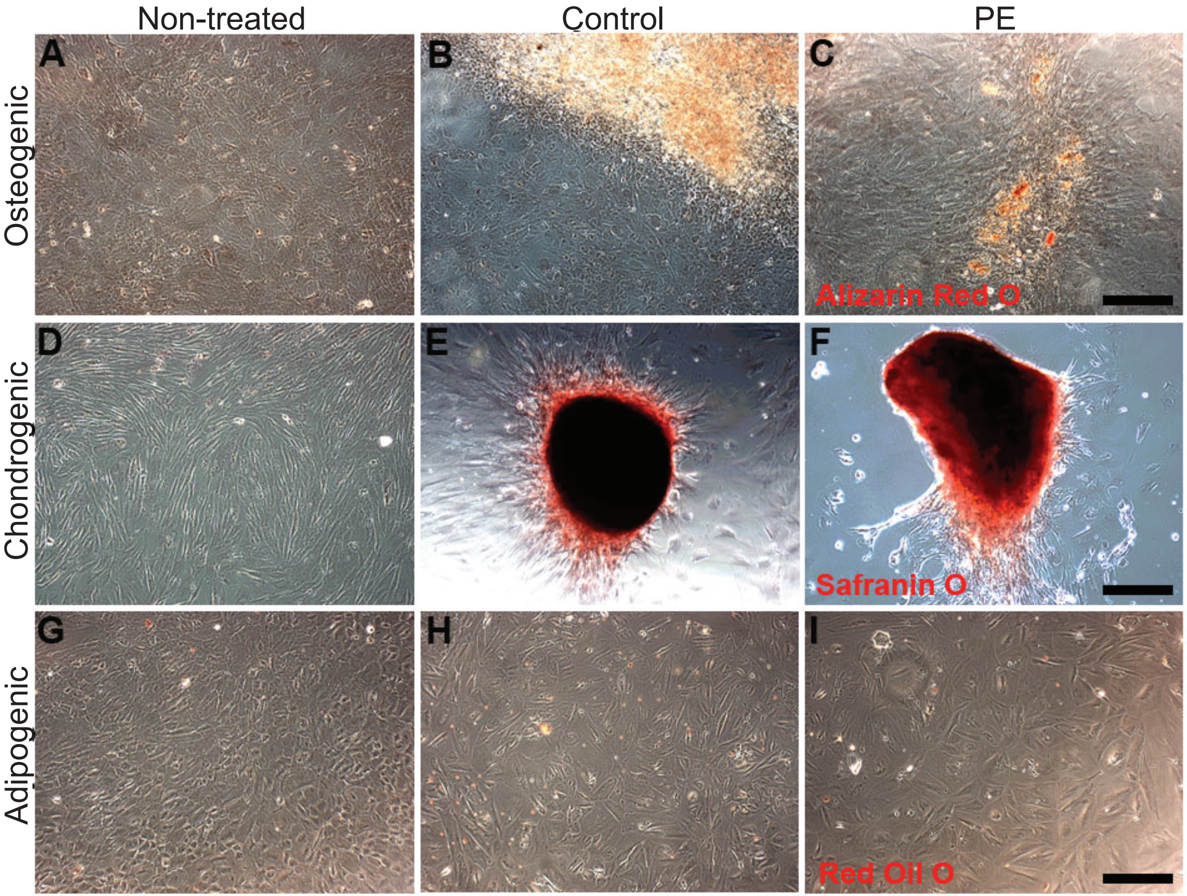

Supplementary Figure 2

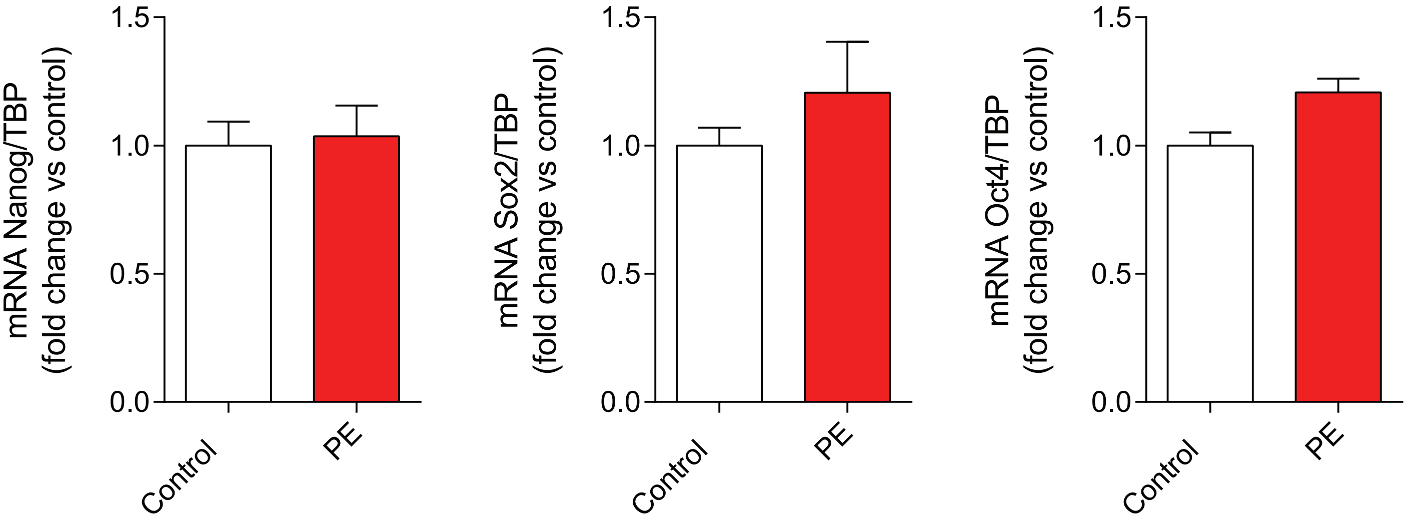

Supplementary Figure 3

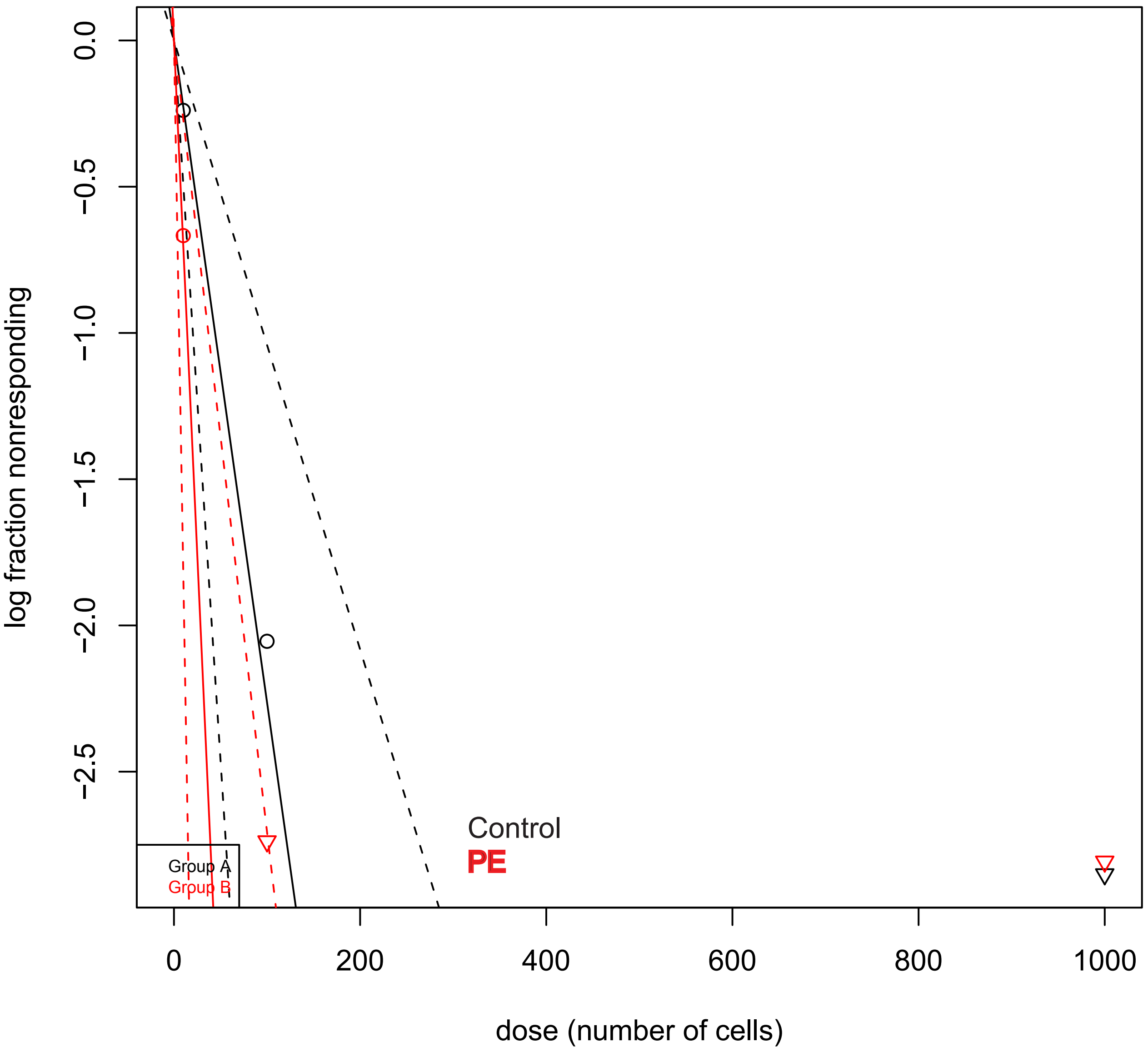

Supplementary Figure 4

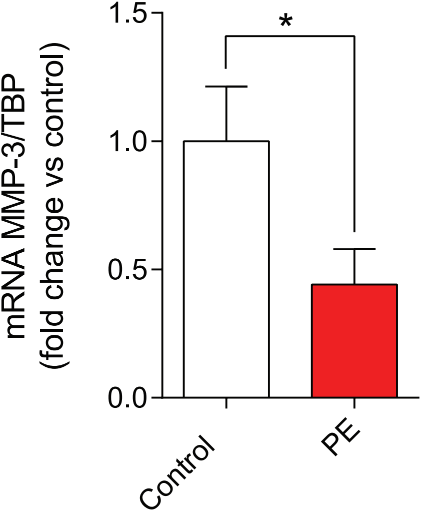

Supplementary Figure 5

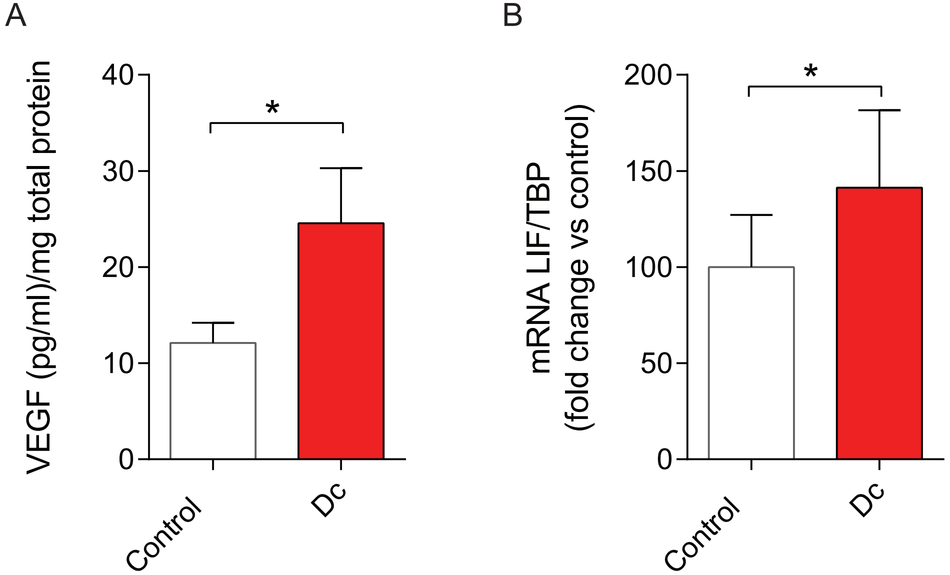

Supplementary Figure 6

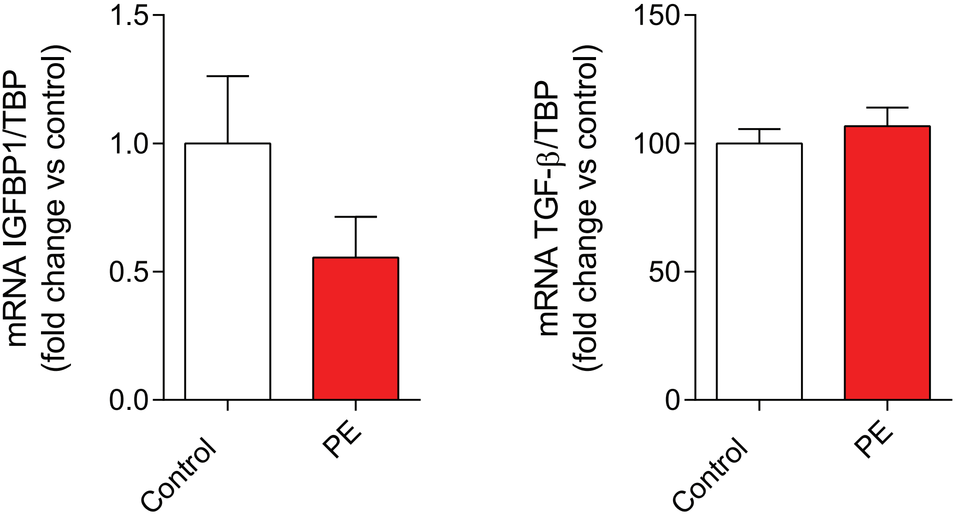

Supplement: Supplementary Materials — Supplementary Figures_Final: file with the legends of each Supplementary Figures. Supplementary Material_Final: file with all the methods related to the experiments performed for the Supplementary Figures. Supplementary Figure 1, 2, 3, 4, 5 and 6: supplementary figures in EPS format. The description of each figure is in the Supplementary Figures_Final file. [file 1916542.f1.pdf]
